# Association Between Neutrophil‐Percentage‐To‐Albumin Ratio Level and Heart Failure in Hypertensive Population

**DOI:** 10.1155/ijhy/9214764

**Published:** 2026-06-19

**Authors:** Mingchen Sun, Zhiyu Li, Zihan Zhou, Jianping Chen, Wei Jia

**Affiliations:** ^1^ College of Medicine, Jishou University, Jishou, Hunan, China, jsu.edu.cn; ^2^ Department of Emergency Medicine, Taizhou Hospital of Zhejiang Province, Taizhou, Zhejiang, China, zjtzyy.h.51daifu.com

**Keywords:** heart failure (HF), hypertension, inflammation, neutrophil percentage-to-albumin ratio (NPAR)

## Abstract

The neutrophil‐to‐albumin ratio (NPAR), which represents a surrogate sign of systemic inflammation, has recently garnered interest as a novel prognostic biomarker in cardiovascular pathology, with a particular focus on heart failure (HF) in patients diagnosed with hypertension. The present analysis explored the relationship between NPAR and HF prevalence based on cross‐sectional data derived from the 2017–2020 cycle of the National Health and Nutrition Examination Survey (NHANES). A total of 3045 hypertensive adults were analyzed, and multivariable logistic regression was employed with sequential adjustment for a comprehensive range of demographic, lifestyle, and clinical variables, including age, sex, ethnicity, body mass index, smoking status, alcohol consumption, diabetes, coronary artery disease, previous myocardial infarction, and history of stroke. The overall weighted prevalence of HF in this hypertensive cohort was 6.27%, and participants with HF had significantly higher NPAR levels than those without HF. After comprehensive adjustment for potential confounders, elevated NPAR remained independently associated with greater odds of HF (OR = 1.17, 95% CI: 1.09–1.25, *p* < 0.001). Additional quartile, blood pressure–adjusted sensitivity, and spline analyses further supported the robustness and approximately linear nature of this association. ROC analysis indicated that NPAR had acceptable discriminatory ability for HF, with an AUC of 0.649 and an optimal cutoff value of 14.24. Subgroup analyses further identified notable interaction effects for alcohol use and stroke history (interaction *p* < 0.05), indicating potential effect modification. These findings suggest that NPAR may serve as an accessible and cost‐effective marker for HF risk stratification in hypertensive individuals.

## 1. Introduction

Hypertension continues to be a major cause of cardiovascular disease (CVD) morbidity and mortality in the United States, affecting approximately one‐third to nearly half of U.S. adults [1]. The widespread prevalence of hypertension imposes substantial burdens on both individual health and the healthcare system. Hypertension is a key etiological factor in the pathogenesis of heart failure (HF), a complex clinical condition frequently regarded as the terminal manifestation of various CVDs. Chronic high blood pressure leads to progressive myocardial damage, reduced exercise capacity, and an elevated risk of adverse cardiac events [2, 3]. Even with advances in therapy, HF patients continue to experience high rates of mortality and hospital readmission, highlighting the need for novel and practical prognostic markers grounded in biology to improve patient outcomes.

The neutrophil‐to‐albumin ratio (NPAR), a marker that combines neutrophil activity and albumin status, is gaining popularity due to its ability to reflect systemic inflammatory burden [4, 5]. Neutrophils, as essential effectors of intrinsic immunity, are typically increased during acute and chronic disease processes [6]. In contrast, lower serum albumin concentrations often indicate ongoing inflammation and poor nutritional status [7]. Together, these two parameters provide a combined metric of an individual’s inflammatory and nutritional state. Several studies have linked elevated NPAR to adverse cardiovascular outcomes, including HF‐related mortality in hypertensive populations [8–10]. Nevertheless, the link between this inflammation‐related biomarker and HF prevalence has not been thoroughly elucidated. Accordingly, this study seeks to find out the cross‐sectional relationship between this inflammatory indicator and HF among hypertensive adults drawn from a demographically diverse U.S. population‐based cohort. This investigation ultimately aims to facilitate timely identification of HF and refine risk assessment strategies within standard clinical settings.

## 2. Methods

### 2.1. Data Source

Data for this analysis were obtained from the National Health and Nutrition Examination Survey (NHANES), a large‐scale surveillance system employing a multistage, cross‐sectional sampling strategy to assess health and nutrition in the U.S. civilian population. NHANES utilizes a complex sampling design involving multistage and stratified probability selection, which supports the extrapolation of the findings to the national U.S. population. Approval for the research procedures was obtained from the NCHS Institutional Review Board, and each participant provided documented consent prior to participation.

### 2.2. Study Population

**FIGURE 1:**
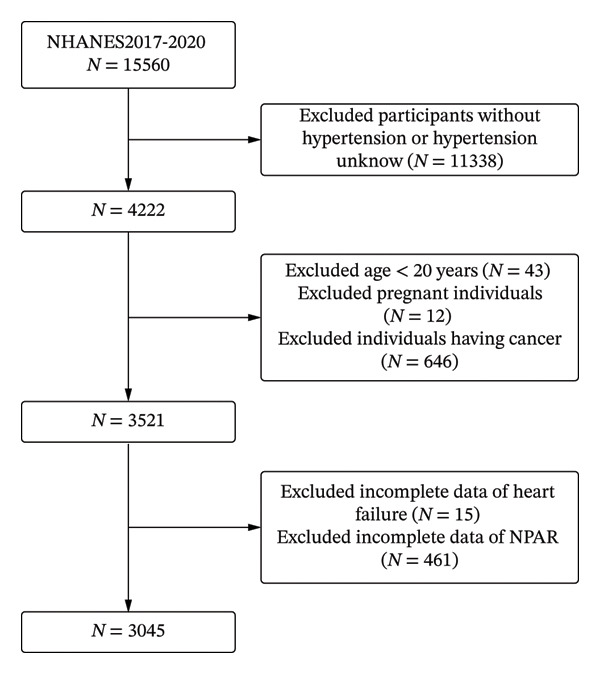
Flow diagram illustrating the participant’s selection procedure for the ultimate analytic sample.

We used NHANES data from the 2017–2020 survey cycles. Out of 15,560 participants initially identified, individuals were excluded if they did not have a confirmed hypertension diagnosis or had indeterminate hypertension status, were under 20 years old, were pregnant, had any malignancy, or were missing data on neutrophil percentage, serum albumin, or HF status. As depicted in Figure 1, 3045 individuals with a confirmed diagnosis of hypertension were identified and included in the final analytic cohort after applying all exclusion criteria.

Hypertension was identified according to any of the following conditions: a self‐reported medical diagnosis, active use of blood pressure–lowering medications, or elevated systolic (≥ 140 mmHg) or diastolic blood pressure (DBP) (≥ 90 mmHg), calculated as the average of three consecutive measurements.

### 2.3. HF

The primary study outcome was HF status, determined through participants’ responses to a standardized NHANES survey question that asked whether a healthcare provider had ever diagnosed them with congestive HF.

### 2.4. Related Variables

The NPAR was calculated by multiplying the neutrophil percentage by 100 and dividing the result by the serum albumin concentration (g/dL) measured from the matched blood sample [11].

A comprehensive set of covariates was incorporated to minimize residual confounding. Covariates were selected a priori based on clinical relevance, evidence from previous literature, and their potential role as confounders in the association between inflammatory markers and HF. Demographic characteristics, cardiovascular risk factors, and comorbid conditions known to influence inflammation or cardiovascular outcomes were included. Sociodemographic characteristics encompassed sex (male/female), race (Mexican American, non‐Hispanic White, Black, Asian, or other), age, marital status (cohabiting/married, separated/divorced/widowed, or never married), and educational attainment (< high school, high school graduate, or > high school). Economic standing was represented using the poverty‐income ratio (PIR: ≤ 1.3, 1.3–3.5, > 3.5), while nutritional status was captured through body mass index (BMI: < 25, 25–30, > 30 kg/m^2^). Lifestyle‐related variables included smoking behavior (never, former, and current) and alcohol intake (none, light/moderate, and heavy) [12]. Medical history variables included self‐reported coronary heart disease, previous myocardial infarction, stroke, diabetes mellitus [13], and hypercholesterolemia [14]. All listed variables were modeled as covariates in multivariable analyses to strengthen estimate validity.

### 2.5. Statistical Analysis

NHANES‐specific sampling weights were applied in all statistical procedures to accommodate the survey’s complex multistage design and ensure the derivation of estimates generalizable to the U.S. population. Descriptive statistics were calculated as weighted means (±standard errors) for continuous variables and weighted percentages for categorical variables. To address incomplete data, multiple imputations by chained equations (MICE) with five imputations were performed to reduce potential bias related to missing data.

Group differences based on HF status were assessed using survey‐weighted Student’s *t*‐tests for continuous variables and Rao–Scott adjusted chi‐square tests for categorical variables. Logistic regression was employed to assess the relationship between inflammatory marker and HF status. Four models were constructed with increasing adjustment: Model I had no covariate adjustment, Model II adjusted for demographic factors, Model III additionally adjusted for behavioral factors, and Model IV further adjusted for clinical comorbidities.

To further examine the association, NPAR was additionally categorized into quartiles, and multivariable logistic regression was performed using the lowest quartile as the reference group. A trend test across quartiles was also conducted by entering the quartile variable as an ordinal term in the regression model.

Sensitivity analyses were performed by additionally adjusting for systolic blood pressure (SBP) and DBP in the fully adjusted model to evaluate whether differences in blood pressure levels within the hypertensive population materially influenced the observed association.

Restricted cubic spline (RCS) functions were incorporated into a fully adjusted logistic regression model to characterize potential nonlinear relationships between the inflammatory biomarker and HF status. In addition, an exploratory threshold effect analysis was conducted using a two‐piecewise logistic regression model to assess whether a potential inflection point existed.

Receiver operating characteristic (ROC) curve analysis was performed to assess the discriminatory ability of NPAR for HF. The area under the ROC curve (AUROC), 95% confidence interval (CI), optimal cutoff value, sensitivity, and specificity were calculated. The optimal cutoff value was determined using the Youden index. The discriminatory performance of NPAR alone and the multivariable model was further compared.

Effect modification was assessed via stratified subgroup analyses, and interaction terms were included to test for statistically significant interactions.

The study outcomes were expressed as adjusted odds ratios (ORs) along with 95% CIs. A two‐sided *p* value of < 0.05 was considered statistically significant. Analytical procedures were implemented using R software (v4.3.3; R Foundation for Statistical Computing, Vienna, Austria) and Free Statistics Software v2.0—both widely accepted open‐source tools in epidemiological analyses.

## 3. Results

### 3.1. Characteristics of Participants

A weighted overview of baseline characteristics for participants enrolled in the 2017–2020 NHANES cycle is presented in Table 1, categorized by HF status. Of the 3045 hypertensive individuals included in the final analytic sample, 191 (6.27%) reported a history of HF. Participants in the HF group were older on average (mean age: 65.3 years) and predominantly male (61.8%), whereas individuals without HF (*n* = 2854) had a younger mean age of 57.8 years and a nearly equal sex distribution.

**TABLE 1:** Characteristics of the study population, NHANES 2017–2020.03.

Variables	Total (*n* = 3045)	Non‐HF	HF	*p*
		(*n* = 2854)	(*n* = 191)	
Gender, *n* (%)				0.002
Male	1550 (50.9)	1432 (50.2)	118 (61.8)	
Female	1495 (49.1)	1422 (49.8)	73 (38.2)	
Race, *n* (%)				0.003
Mexican American	280 (9.2)	10 (5.2)	270 (9.5)	
Non‐Hispanic White	996 (32.7)	76 (39.8)	920 (32.2)	
Non‐Hispanic Black	1012 (33.2)	74 (38.7)	938 (32.9)	
Non‐Hispanic Asian	308 (10.1)	9 (4.7)	299 (10.5)	
Other	449 (14.7)	22 (11.5)	427 (15)	
Age(years)	58.3 ± 14.3	57.8 ± 14.4	65.3 ± 11.5	< 0.001
Marital status, *n* (%)				0.002
Married/living with partner	1693 (55.7)	1594 (55.9)	99 (51.8)	
Widowed/divorced/separated	922 (30.3)	845 (29.6)	77 (40.3)	
Never married	426 (14.0)	411 (14.4)	15 (7.9)	
PIR, *n* (%)				< 0.001
≤ 1.3	777 (29.8)	720 (29.5)	57 (35.2)	
1.3–3.5	1054 (40.5)	973 (39.8)	81 (50)	
> 3.5	774 (29.7)	750 (30.7)	24 (14.8)	
Education level, *n* (%)				< 0.001
< High school	272 (8.9)	251 (8.8)	21 (11)	
High school	1180 (38.8)	1084 (38)	96 (50.3)	
> High school	1588 (52.2)	1514 (53.1)	74 (38.7)	
BMI, *n* (%)				0.046
< 25	506 (17.0)	482 (17.2)	24 (13)	
25–30	906 (30.4)	859 (30.7)	47 (25.5)	
> 30	1570 (52.6)	1457 (52.1)	113 (61.4)	
Smoking, *n* (%)				< 0.001
Never	1597 (52.5)	1525 (53.5)	72 (37.7)	
Ever	857 (28.2)	773 (27.1)	84 (44)	
Current	589 (19.4)	554 (19.4)	35 (18.3)	
Diabetes, *n* (%)				< 0.001
Yes	895 (29.4)	799 (28)	96 (50.3)	
No	2150 (70.6)	2055 (72)	95 (49.7)	
Alcohol consumption, *n* (%)				< 0.001
Nondrinker	1542 (59.1)	1420 (58.1)	122 (73.5)	
Low to moderate drinker	135 (5.2)	125 (5.1)	10 (6)	
Heavy drinker	931 (35.7)	897 (36.7)	34 (20.5)	
NPAR	14.4 ± 2.9	14.3 ± 2.9	15.9 ± 3.3	< 0.001
Coronary heart disease, *n* (%)				< 0.001
Yes	205 (6.8)	128 (4.5)	77 (41)	
No	2828 (93.2)	2717 (95.5)	111 (59)	
High cholesterol, *n* (%)				0.151
Yes	2497 (82.0)	164 (85.9)	2333 (81.7)	
No	548 (18.0)	27 (14.1)	521 (18.3)	
History MI, *n* (%)				< 0.001
Yes	222 (7.3)	140 (4.9)	82 (43.4)	
No	2816 (92.7)	2709 (95.1)	107 (56.6)	
Stroke, *n* (%)				< 0.001
Yes	257 (8.5)	210 (7.4)	47 (24.7)	
No	2782 (91.5)	2639 (92.6)	143 (75.3)	
SBP (mmHg)	135.7 ± 19.9	135.9 ± 19.6	131.9 ± 23.1	0.01
DBP (mmHg)	79.6 ± 12.9	80.0 ± 12.8	73.8 ± 13.0	< 0.001

Participants with HF exhibited significantly different demographic, behavioral, and clinical characteristics compared to those without HF, with all between‐group comparisons demonstrating statistical significance at the *p* < 0.05 level. In contrast, no significant variation was noted in the prevalence of hypercholesterolemia.

### 3.2. Multivariable Analysis Linking NPAR to HF Risk

As shown in Table 2, the stepwise‐adjusted logistic regression models revealed a consistent trend, whereby elevated NPAR levels were related to an increased likelihood of HF. In Model I, which was the basic model, each unit increase in NPAR corresponded to an OR of 1.19 (95% CI: 1.14–1.25; *p* < 0.05) for HF. This relationship remained basically similar after correcting for demographics in Model II (OR: 1.19, 95% CI: 1.13–1.25), adding behavioral factors in Model III (OR: 1.18, 95% CI: 1.11–1.25), and further including clinical comorbidities in Model IV (OR: 1.17, 95% CI: 1.09–1.25). The NPAR–HF correlation was statistically significant in all models (*p* < 0.05), indicating an independent and stable relationship between higher NPAR and HF risk.

**TABLE 2:** Multivariate regression analysis of the association between NPAR and HF.

Variable	OR (95% CI)	*p*
Model I	1.19 (1.14∼1.25)	< 0.001
Model II	1.19 (1.13∼1.25)	< 0.001
Model III	1.18 (1.11∼1.25)	< 0.001
Model IV	1.17 (1.09∼1.25)	< 0.001

*Note:* Model I: no adjusted. Model II: adjusted for gender + race + age. Model III: Model II + marital status + PIR + education level + BMI. Model IV: Model III + smoking + diabetes + alcohol consumption + coronary heart disease + high cholesterol + history MI + stroke.

### 3.3. Association Between NPAR Quartiles and HF

When NPAR was analyzed in quartiles, a graded positive association with HF was observed. Compared with participants in the lowest quartile, those in the third quartile (OR: 1.84, 95% CI: 1.05–3.24) and fourth quartile (OR: 2.75, 95% CI: 1.60–4.70) had significantly higher odds of HF after multivariable adjustment. A significant trend across quartiles was identified (*p* for trend < 0.001), as shown in Table 3.

**TABLE 3:** Association between NPAR quartiles and heart failure.

NPAR quartiles	Adjusted OR (95% CI)	*p*
Q1 (reference)	1.00 (reference)	
Q2	1.16 (0.64–2.10)	0.634
Q3	1.84 (1.05–3.24)	0.033
Q4	2.75 (1.60–4.70)	< 0.001
*p* for trend		< 0.001

### 3.4. Sensitivity Analyses Additionally Adjusting for Blood Pressure Levels

In sensitivity analyses, the association between NPAR and HF remained stable after additional adjustment for blood pressure levels. Further adjustment for SBP (OR: 1.16, 95% CI: 1.10–1.23, *p* < 0.001) and for both SBP and DBP (OR: 1.16, 95% CI: 1.09–1.23, *p* < 0.001) yielded similar results, supporting the robustness of the main findings (Table 4).

**TABLE 4:** Sensitivity analyses additionally adjusting for SBP and DBP.

Variable	OR (95% CI)	*p*
Main model (Model IV)	1.17 (1.09–1.25)	< 0.001
Model IV + SBP	1.16 (1.10–1.23)	< 0.001
Model IV + SBP + DBP	1.16 (1.09–1.23)	< 0.001

### 3.5. RCS and Threshold Effect Analyses

**FIGURE 2:**
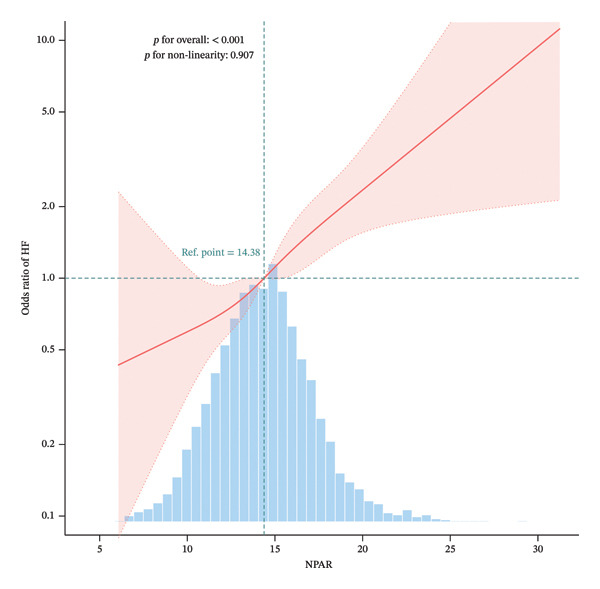
Restricted cubic spline analysis of the association between NPAR and heart failure. The fully adjusted model treated NPAR as a continuous variable and applied penalized spline smoothing to test for nonlinearity. The median NPAR value is the reference point. Shaded areas indicate 95% CIs. The plot shows a steady increase in HF risk with rising NPAR, especially around the median range. Widening CIs at the extremes of NPAR reflect fewer observations and the nature of spline‐based estimation.

To explore the relationship between inflammatory markers and the likelihood of HF, a RCS approach was employed within a comprehensively adjusted logistic model. As shown in Figure 2, the analysis revealed a consistent linear increase in risk without evidence of deviation from linearity (*p* for nonlinearity = 0.907). In exploratory threshold effect analysis, a potential breakpoint was identified at 14.86; however, the two‐piecewise logistic regression model did not provide a significantly better fit than the linear model (*p* = 0.923), indicating no clear threshold effect. The detailed threshold effect results are presented in Supporting Table S1.

### 3.6. ROC Analysis of NPAR for Identifying HF

**FIGURE 3:**
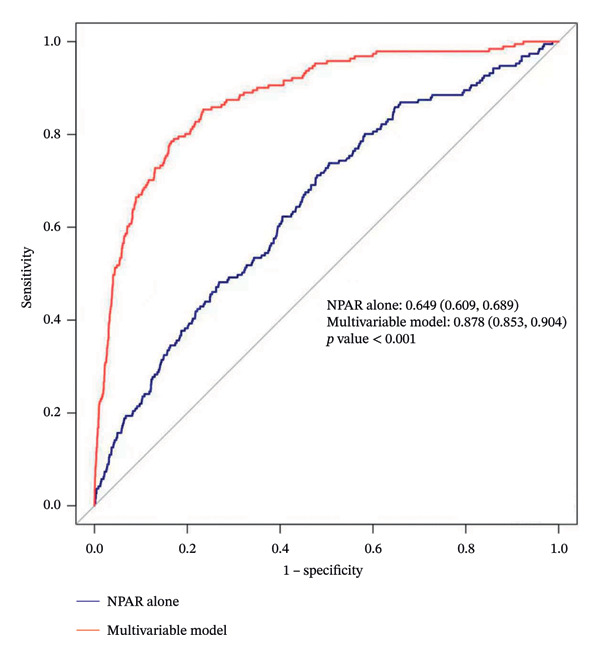
Receiver operating characteristic (ROC) curves comparing the discriminatory performance of NPAR alone and the multivariable model for heart failure. The blue curve represents NPAR alone, and the red curve represents the multivariable model. The multivariable model showed significantly better discrimination than NPAR alone (AUC: 0.878 vs. 0.649, *p* < 0.001).

ROC analysis showed that NPAR alone had modest discriminatory ability for HF, with an AUC of 0.649 (95% CI: 0.609–0.689). The optimal cutoff value of NPAR was 14.24, corresponding to a sensitivity of 0.738 and a specificity of 0.495. In contrast, the multivariable model demonstrated substantially better discrimination, with an AUC of 0.878 (95% CI: 0.853–0.904), and the difference between the two ROC curves was statistically significant (*p* < 0.001). Figure 3 illustrates the ROC curves, and the detailed diagnostic indices are summarized in Supporting Table S2.

### 3.7. Relationships Between NPAR and HF in Subgroups

**FIGURE 4:**
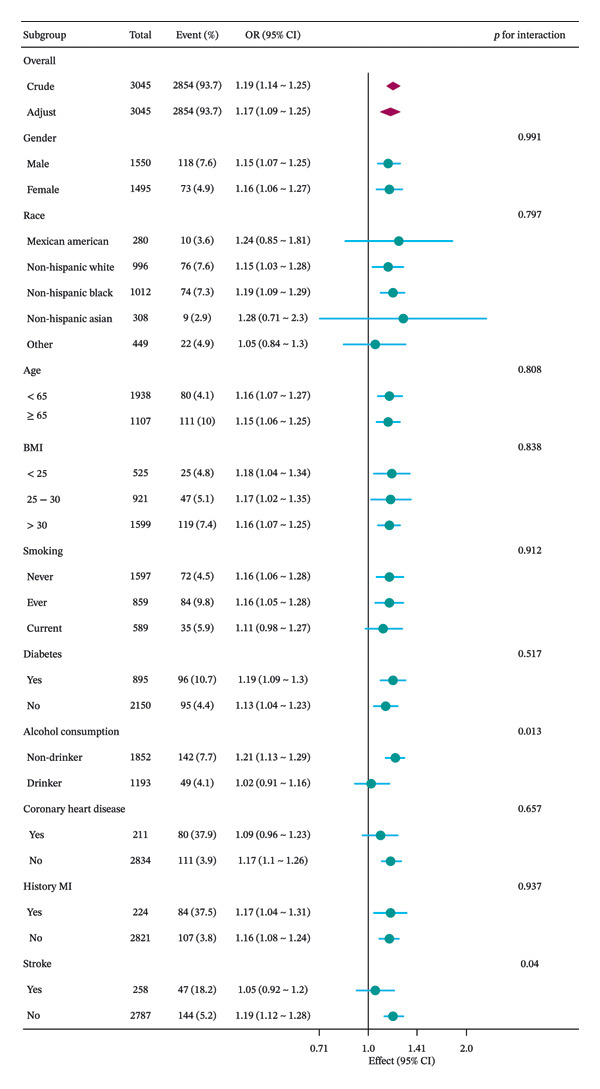
Associations between NPAR and heart failure stratified by clinical subgroups. Models were adjusted for all relevant covariates. Interaction terms were tested to identify subgroup‐specific variations in effect size. ORs and 95% CIs are shown for each category.

Figure 4 presents the results of stratified analyses conducted to evaluate the robustness of the association between NPAR and HF across multiple subgroups. Due to the limited number of participants categorized as low‐to‐moderate alcohol users, alcohol intake was dichotomized as either “nondrinker” or “drinker” for analysis.

Overall, the positive association between NPAR and HF remained generally consistent after full adjustment for covariates. Stronger associations were particularly observed among non‐Hispanic Whites, non‐Hispanic Blacks, never and ever smokers, nondrinkers, and individuals without coronary heart disease or prior stroke.

Notably, statistically significant interaction effects were identified for alcohol consumption (*p* for interaction = 0.013) and prior stroke history (*p* for interaction = 0.04). Among nondrinkers, each unit increase in NPAR was associated with 21% higher odds of HF (OR = 1.21, 95% CI: 1.13–1.29), whereas no significant association was observed among drinkers (OR = 1.02, 95% CI: 0.91–1.16). Similarly, in participants without a history of stroke, NPAR was significantly associated with HF (OR = 1.19, 95% CI: 1.12–1.28), whereas the association was attenuated and nonsignificant among those with prior stroke (OR = 1.05, 95% CI: 0.92–1.20).

These findings indicate that the strength of the NPAR–HF association varies according to alcohol consumption and stroke history, supporting the presence of effect modification. Further investigation into the biological and clinical mechanisms underlying these differences may help refine risk stratification strategies in hypertensive populations.

## 4. Discussion

In this cross‐sectional analysis of a nationally representative hypertensive population, higher NPAR levels were independently associated with greater odds of HF after extensive adjustment for potential confounders. This association remained stable after additional adjustment for blood pressure levels, supporting the robustness of the main findings. When NPAR was further analyzed in quartiles, participants in the higher quartiles showed markedly increased odds of HF, with a significant dose–response trend across quartiles. RCS analysis suggested an approximately linear positive association between NPAR and HF, and exploratory threshold effect analysis did not support a clear nonlinear or threshold relationship. ROC analysis showed that NPAR alone had modest discriminatory ability for HF, whereas discrimination was substantially improved in the multivariable model. We also found that the association between NPAR and HF varied according to alcohol use and stroke history, suggesting that these clinical factors may modify the inflammatory and vascular context in which NPAR reflects HF susceptibility in hypertensive individuals.

Hypertension provides a specific pathophysiological substrate for HF development. Chronic elevation of arterial pressure imposes sustained left ventricular pressure overload and may be accompanied by intravascular volume expansion, which together initiate maladaptive cardiac remodeling [15]. Early stages are characterized by concentric left ventricular hypertrophy and increased myocardial stiffness with progressive diastolic dysfunction; over time, persistent hemodynamic stress and neurohormonal overactivation (including the renin–angiotensin–aldosterone system (RAAS) and sympathetic nervous system) promote oxidative stress, inflammatory signaling, and extracellular matrix remodeling, thereby accelerating myocardial fibrosis and impairing both relaxation and contractile efficiency [15–17]. In parallel, increased arterial stiffness and microvascular endothelial dysfunction reduce tissue perfusion and further amplify afterload, creating a vicious cycle that facilitates transition from hypertensive heart disease to overt HF [17]. Within this hypertensive milieu, inflammation is increasingly recognized not merely as an epiphenomenon but as an active mediator that can exacerbate vascular injury and adverse myocardial remodeling, providing biological plausibility for inflammation‐based indices such as NPAR to capture heightened HF susceptibility in hypertensive individuals [15, 16, 18].

Our findings align with an increasing amount of evidence implicating systemic inflammation as a key contributor to HF pathogenesis. For example, as a prototypical inflammatory biomarker, C‐reactive protein (CRP) has demonstrated a robust correlation with heightened cardiovascular risk across multiple prior studies [19]. Epidemiological data further indicate that elevated CRP levels can predict the development of HF. Supporting this, a Mendelian randomization analysis revealed that genetically raised CRP levels were connected to a 21% greater likelihood of hypertensive heart disease, independent of conventional cardiovascular risk factors [20]. Similarly, NPAR—an inflammation‐based index derived from routine laboratory measures—has demonstrated a prognostic value in various clinical settings. Prior research has found that higher NPAR is associated with greater all‐cause mortality in patients with established HF, coupled with increased cardiovascular and all‐cause mortality in hypertensive populations [8, 21]. These findings emphasize the possible value of inflammation‐related biomarkers, such as CRP and NPAR, in enhancing cardiovascular risk stratification, thereby supporting the biological plausibility of the observed association between elevated NPAR and prevalent HF.

NPAR integrates two biologically complementary signals: neutrophil‐driven inflammatory activation and reduced albumin‐related protective capacity [22, 23]. In hypertensive individuals, this composite profile may reflect an inflammatory burden superimposed on an already vulnerable cardiovascular substrate, thereby contributing to HF development.

In hypertension, activated neutrophils contribute to endothelial damage and vascular remodeling—both precursors of hypertensive heart disease [22]. The production of neutrophil extracellular traps (NETs)—networks of DNA, histones, and granule components—represents a pivotal immune process that, despite its protective role, may trigger microvascular occlusion, thrombogenesis, and endothelial dysfunction [24, 25]. NET‐derived factors such as reactive oxygen species (ROS) and proteolytic enzymes further exacerbate oxidative damage and promote apoptotic cell death, ultimately leading to myocardial fibrosis and pathological cardiac remodeling [26–28]. Moreover, NETs have been implicated in the stimulation of the RAAS, promoting increased blood vessel tone and enhanced sodium reabsorption—two key processes that lead to the advancement of HF in hypertensive individuals [29].

Albumin, a liver‐synthesized plasma protein, contributes to vascular homeostasis through its roles in maintaining oncotic pressure, regulating capillary permeability, and supporting endothelial function [23]. In the context of hypertension, hypoalbuminemia is frequently encountered and reflects persistent inflammation and latent organ dysfunction, both of which are exacerbated in HF [30]. An elevated NPAR thus signals a combination of intensified inflammatory response and compromised albumin‐mediated vascular protection [31]. Reduced serum albumin has been associated with increased endothelial leakage, fluid imbalance, and diminished anti‐inflammatory modulation, all of which are implicated in hallmark HF symptoms such as pulmonary congestion and edema [32]. Beyond osmotic balance, albumin inhibits cytokine release and modulates leukocyte‐endothelial crosstalk [33, 34]; when levels fall, this regulatory capacity is impaired, perpetuating vascular inflammation and myocardial dysfunction. This biological context supports the role of NPAR as a meaningful indicator of HF risk among hypertensive populations.

The association between NPAR and HF in hypertensive individuals may reflect the convergence of inflammatory activation with the structural, microvascular, and hemodynamic abnormalities that drive the progression from hypertensive heart disease to overt HF [15, 35]. Chronic hypertension induces left ventricular hypertrophy, interstitial and perivascular fibrosis, endothelial and coronary microvascular dysfunction, and impaired myocardial relaxation, all of which progressively increase ventricular stiffness and left‐sided filling pressures. These abnormalities promote left atrial remodeling, pulmonary venous hypertension, and congestion, thereby providing the structural and hemodynamic substrate for overt HF [15, 17, 35]. Within this setting, a higher neutrophil proportion may reflect intensified innate immune activation, oxidative stress, and NET‐related endothelial and microvascular injury, which can further aggravate profibrotic remodeling and diastolic dysfunction [25, 28]. At the same time, lower albumin may indicate reduced vascular‐protective reserve, persistent low‐grade inflammation, and impaired endothelial homeostasis; once filling pressures rise, this state may also favor clinical decompensation rather than directly impair myocardial contractility [23, 36]. Accordingly, an elevated NPAR may identify hypertensive individuals in whom inflammatory microvascular injury, myocardial stiffening, and reduced compensatory reserve act together to facilitate the transition from hypertensive cardiac remodeling to clinical HF [35, 36].

From a clinical perspective, NPAR may offer several practical advantages over more resource‐intensive assessment approaches because it is derived from two routinely available laboratory parameters, neutrophil percentage and serum albumin, without requiring additional testing, imaging, or specialized assays. In the present study, ROC analysis showed that NPAR alone had only modest discriminatory ability for HF, indicating that it should not be considered a stand‐alone diagnostic tool. However, its simplicity, low cost, and wide availability may still make it useful as an adjunctive marker for preliminary risk stratification in hypertensive populations, particularly in primary care or resource‐limited settings. When interpreted together with conventional clinical characteristics, NPAR may help identify individuals who warrant closer cardiovascular evaluation or follow‐up.

**FIGURE 5:**
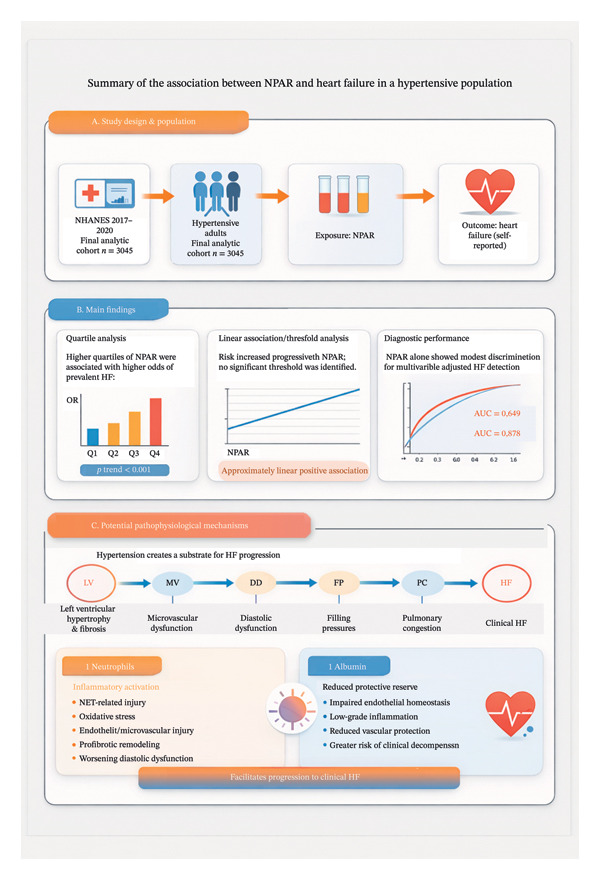
Graphical summary of the association between NPAR and heart failure in a hypertensive population. The figure illustrates the study design, principal findings, and proposed biological interpretation. Higher NPAR levels were associated with increased odds of heart failure, with a significant quartile trend and an approximately linear positive association. The lower panel summarizes the proposed mechanisms linking neutrophil‐related inflammatory activation and reduced albumin‐related protective reserve to heart failure progression in hypertensive individuals.

A graphical summary of the study design, main findings, and proposed biological interpretation is provided in Figure 5.

Nevertheless, there are several limitations. Firstly, owing to the cross‐sectional design, causal relationships between the inflammation markers and the development of HF cannot be established, and temporal sequencing remains unclear. Because HF status reflects a history of physician‐diagnosed disease and NPAR was measured at the examination visit, the temporal sequence between inflammatory status and HF onset cannot be determined. Secondly, while we adjusted for several confounders, unmeasured or residual confounding may still exist. Thirdly, HF status was ascertained by self‐report, which could introduce recall bias or misclassification. Finally, we lacked mechanistic biomarkers and longitudinal follow‐up data, which limit our ability to explore causal pathways and to assess the long‐term prognostic value of NPAR.

## 5. Conclusions

A significant linear relationship was observed between NPAR levels and the likelihood of HF among individuals with hypertension. Elevated NPAR values were further associated with an increased susceptibility to HF in this population. Our data also suggested potential interactions between alcohol consumption or stroke and NPAR, which may impact the link between NPAR and HF. These results provide supporting evidence for monitoring and managing NPAR levels in hypertensive patients to lower the risk of HF.

## Author Contributions

Mingchen Sun contributed to study planning, data analysis, and manuscript drafting. Wei Jia contributed to study planning and manuscript development. Jianping Chen, Zhiyu Li, and Zihan Zhou contributed to manuscript review.

## Funding

This research was funded by the Doctoral Scientific Research Project of Jishou University (Project No. 201811).

## Disclosure

All authors contributed to the development of the manuscript and approved the final version for submission.

## Ethics Statement

The NHANES protocols were reviewed and approved by the Research Ethics Review Board of the National Center for Health Statistics (NCHS) under Protocol #2018‐01. All participants provided written informed consent prior to participation. The present analysis was based on deidentified, publicly accessible data from the 2017–2020 NHANES cycles and was therefore exempted from additional institutional ethical review.

## Conflicts of Interest

The authors declare no conflicts of interest.

## Supporting Information

Additional supporting information can be found online in the Supporting Information section.

## Supporting information


**Supporting Information 1** Supporting File 1: STROBE Checklist for Cross‐Sectional Studies. This checklist is provided to demonstrate compliance with the Strengthening the Reporting of Observational Studies in Epidemiology (STROBE) reporting guidelines for cross‐sectional studies and to facilitate editorial assessment of reporting completeness.

**Supporting Information 2** The supporting material for this study includes two supporting tables. Supporting Table S1 presents the exploratory threshold effect analysis of the association between neutrophil‐percentage‐to‐albumin ratio (NPAR) and heart failure, including the estimated breakpoint, odds ratios below and above the breakpoint, and the comparison between the two‐piecewise logistic regression model and the linear model. Supporting Table S2 presents the diagnostic performance indices of NPAR for identifying prevalent heart failure, including the area under the receiver operating characteristic curve (AUC), optimal cutoff value, sensitivity, and specificity.

## Data Availability

The datasets analyzed in this study are derived from publicly accessible resources and can be retrieved through official online repositories. Detailed access information is available from the corresponding author upon reasonable request.
