# Supplementary material for: Association Between Neutrophil‐Percentage‐To‐Albumin Ratio Level and Heart Failure in Hypertensive Population
Source: Int J Hypertens. 2026 Jun 19;2026:9214764. doi: 10.1155/ijhy/9214764 (PMC13282547; doi:10.1155/ijhy/9214764)
Supplement: Supplementary file 2 — Supporting Information 2 The supporting material for this study includes two supporting tables. Supporting Table S1 presents the exploratory threshold effect analysis of the association between neutrophil‐percentage‐to‐albumin ratio (NPAR) and heart failure, including the estimated breakpoint, odds ratios below and above the breakpoint, and the comparison between the two‐piecewise logistic regression model and the linear model. Supporting Table S2 presents the diagnostic performance indices of NPAR for identifying prevalent heart failure, including the area under the receiver operating characteristic curve (AUC), optimal cutoff value, sensitivity, and specificity. [file IJHY-2026-9214764-s001.pdf]

STROBE-MR checklist of recommended items to address in reports of Mendelian randomization studies[1 2]

Item No.	Section		Checklist item	Page No.	Relevant text from manuscript
1	**TITLE and ABSTRACT**		Indicate Mendelian randomization (MR) as the study's design in the title and/or the abstract if that is a main purpose of the study	1	Abstract: "The neutrophil-to-albumin ratio (NPAR) … explored the relationship between NPAR and HF prevalence based on cross-sectional data …". (Study is not MR; MR-specific reporting not applicable.)
	**INTRODUCTION**				
2	**Background**		Explain the scientific background and rationale for the reported study. What is the exposure? Is a potential causal relationship between exposure and outcome plausible? Justify why MR is a helpful method to address the study question	2	Introduction: Background and rationale for studying NPAR and HF in hypertensive adults; need for practical prognostic markers.
3	**Objectives**		State specific objectives clearly, including pre-specified causal hypotheses (if any). State that MR is a method that, under specific assumptions, intends to estimate causal effects	3	Introduction (Objectives): "This study seeks to evaluate the cross-sectional relationship between NPAR and heart failure among hypertensive adults …".
	**METHODS**				
4	**Study design and data sources**		Present key elements of the study design early in the article. Consider including a table listing sources of data for all phases of the study. For each data source contributing to the analysis, describe the following:	4–6	Methods overview: NHANES 2017–2020, cross-sectional, nationally representative sample.
		a)	Setting: Describe the study design and the underlying population, if possible. Describe the setting, locations, and relevant dates, including periods of recruitment, exposure, follow-up, and data collection, when available.	4–5	Setting: NHANES multistage, stratified sampling; U.S. civilian population; 2017–2020 cycles.
		b)	Participants: Give the eligibility criteria, and the sources and methods of selection of participants. Report the sample size, and whether any power or sample size calculations were carried out prior to the main analysis	5	Participants: Eligibility/exclusions; final analytic cohort n = 3,045 with hypertension; flowchart (Figure 1).
		c)	Describe measurement, quality control and selection of genetic variants	N/A	Not applicable (no genetic variants; non-MR study).

		d)	For each exposure, outcome, and other relevant variables, describe methods of assessment and diagnostic criteria for diseases	5–6	Variables/assessment: NPAR = neutrophil% × 100 / albumin (g/dL); HF from standardized NHANES question; covariates listed with categories.
		e)	Provide details of ethics committee approval and participant informed consent, if relevant	6	Ethics: NCHS IRB approval; informed consent obtained for NHANES.
5	**Assumptions**		Explicitly state the three core IV assumptions for the main analysis (relevance, independence and exclusion restriction) as well assumptions for any additional or sensitivity analysis	N/A	Not applicable (IV assumptions—relevance/independence/exclusion—are MR-specific).
6	**Statistical methods: main analysis**		Describe statistical methods and statistics used	6–7	Statistical methods: survey weights; logistic regression with four adjustment models; subgroup/interaction; restricted cubic spline.
		a)	Describe how quantitative variables were handled in the analyses (i.e., scale, units, model)	6–7	Quantitative variables handled as continuous or categorical per definitions; ORs with 95% CIs; two-sided P<0.05.
		b)	Describe how genetic variants were handled in the analyses and, if applicable, how their weights were selected	N/A	Not applicable (no genetic variants; non-MR study).
		c)	Describe the MR estimator (e.g. two-stage least squares, Wald ratio) and related statistics. Detail the included covariates and, in case of two-sample MR, whether the same covariate set was used for adjustment in the two samples	N/A	Not applicable (no MR estimator used).
		d)	Explain how missing data were addressed	6–7	Missing data addressed via multiple imputation by chained equations (MICE) with five iterations.
		e)	If applicable, indicate how multiple testing was addressed	—	Multiple testing not explicitly applied; primary analyses prespecified.
7	**Assessment of assumptions**		Describe any methods or prior knowledge used to assess the assumptions or justify their validity	N/A	Not applicable (no MR IV assumptions to assess).
8	**Sensitivity analyses and additional analyses**		Describe any sensitivity analyses or additional analyses performed (e.g. comparison of effect estimates from different approaches, independent replication, bias analytic techniques, validation of instruments, simulations)	9–11	Additional analyses: stratified subgroup analyses with interaction tests; restricted

					cubic spline (Figure 2); forest/subgroup plot (Figure 3).
9	**Software and pre-registration**			7	Software: R v4.3.3; Free Statistics Software v2.0; no preregistration stated.
		a)	Name statistical software and package(s), including version and settings used	7	R (v4.3.3) and Free Statistics Software v2.0 used.
		b)	State whether the study protocol and details were pre-registered (as well as when and where)	N/A	Study protocol not pre-registered.
	**RESULTS**				
10	**Descriptive data**			8–9	Descriptive data summarized in Table 1; HF prevalence 6.27% (n=191).
		a)	Report the numbers of individuals at each stage of included studies and reasons for exclusion. Consider use of a flow diagram	5	Numbers at each stage and exclusions; Flow diagram (Figure 1).
		b)	Report summary statistics for phenotypic exposure(s), outcome(s), and other relevant variables (e.g. means, SDs, proportions)	8–9	Baseline characteristics by HF status (Table 1).
		c)	If the data sources include meta-analyses of previous studies, provide the assessments of heterogeneity across these studies	N/A	Not a meta-analysis; not applicable.
		d)	For two-sample MR:<br>i. Provide justification of the similarity of the genetic variant-exposure associations between the exposure and outcome samples<br>ii. Provide information on the number of individuals who overlap between the exposure and outcome studies	N/A	Not a two-sample MR; not applicable.
11	**Main results**			9–11	Main results: higher NPAR associated with increased odds of HF across models; OR≈1.17 (95% CI 1.09–1.25).
		a)	Report the associations between genetic variant and exposure, and between genetic variant and outcome, preferably on an interpretable scale	N/A	Not applicable (no genetic variant associations).
		b)	Report MR estimates of the relationship between exposure and outcome, and the measures of uncertainty from the MR analysis, on an interpretable scale, such as odds ratio or relative risk per SD difference	N/A	Not applicable (no MR estimates).

		c)	If relevant, consider translating estimates of relative risk into absolute risk for a meaningful time period	—	Absolute risk translation not presented.
		d)	Consider plots to visualize results (e.g. forest plot, scatterplot of associations between genetic variants and outcome versus between genetic variants and exposure)	10–11	Figures: RCS plot (Figure 2); subgroup plot (Figure 3).
12	**Assessment of assumptions**			N/A	Not applicable (MR assumption assessments not performed).
		a)	Report the assessment of the validity of the assumptions	N/A	—
		b)	Report any additional statistics (e.g., assessments of heterogeneity across genetic variants, such as I^2, Q statistic or E-value)	N/A	—
13	**Sensitivity analyses and additional analyses**			9–11	Sensitivity/additional analyses: subgroup analyses with interaction terms; spline checks for linearity.
		a)	Report any sensitivity analyses to assess the robustness of the main results to violations of the assumptions	—	Robustness via subgroup/interaction analyses; no MR-specific sensitivity (e.g., MR-Egger) performed.
		b)	Report results from other sensitivity analyses or additional analyses	—	Additional analyses limited to subgroup interactions and spline.
		c)	Report any assessment of direction of causal relationship (e.g., bidirectional MR)	N/A	Not applicable (no bidirectional MR).
		d)	When relevant, report and compare with estimates from non-MR analyses	—	Non-MR observational analyses (primary models) reported.
		e)	Consider additional plots to visualize results (e.g., leave-one-out analyses)	10–11	Additional plots: subgroup forest/interaction plot (Figure 3).
	**DISCUSSION**				
14	**Key results**		Summarize key results with reference to study objectives	12	Key results summarized with reference to objectives (Discussion opening).
15	**Limitations**		Discuss limitations of the study, taking into account the validity of the IV assumptions, other sources of potential bias, and imprecision. Discuss both direction and magnitude of any potential bias and any efforts to address them	15	Limitations: cross-sectional design (no causality), residual confounding, self-reported HF, lack of mechanistic biomarkers/longitudinal data.

16	**Interpretation**			13–14	Interpretation in context of inflammation and prior evidence; careful causal language.
		a)	Meaning: Give a cautious overall interpretation of results in the context of their limitations and in comparison with other studies	13	Meaning: NPAR independently associated with HF in hypertensive adults; linear association by RCS.
		b)	Mechanism: Discuss underlying biological mechanisms that could drive a potential causal relationship between the investigated exposure and the outcome, and whether the gene-environment equivalence assumption is reasonable. Use causal language carefully, clarifying that IV estimates may provide causal effects only under certain assumptions	13–14	Mechanism: inflammation (neutrophils/albumin), NETs, endothelial dysfunction; CRP literature (including MR evidence for CRP).
		c)	Clinical relevance: Discuss whether the results have clinical or public policy relevance, and to what extent they inform effect sizes of possible interventions	14	Clinical relevance: NPAR may aid risk stratification and early identification in routine practice.
17	**Generalizability**		Discuss the generalizability of the study results (a) to other populations, (b) across other exposure periods/timings, and (c) across other levels of exposure	16	Generalizability primarily to U.S. hypertensive adults; applicability to other populations/time windows not directly tested.
	**OTHER INFORMATION**				
18	**Funding**		Describe sources of funding and the role of funders in the present study and, if applicable, sources of funding for the databases and original study or studies on which the present study is based	1	Funding statement provided (Doctoral Scientific Research Project of Jishou University, No. 201811).
19	**Data and data sharing**		Provide the data used to perform all analyses or report where and how the data can be accessed, and reference these sources in the article. Provide the statistical code needed to reproduce the results in the article, or report whether the code is publicly accessible and if so, where	17	Data availability: NHANES public repositories; details available upon request.
20	**Conflicts of Interest**		All authors should declare all potential conflicts of interest	17	Conflict of Interest: authors declare no commercial or financial relationships.

This checklist is copyrighted by the Equator Network under the Creative Commons Attribution 3.0 Unported (CC BY 3.0) license.

1. Skrivankova VW, Richmond RC, Woolf BAR, Yarmolinsky J, Davies NM, Swanson SA, et al. Strengthening the Reporting of Observational Studies in Epidemiology using Mendelian Randomization (STROBE-MR) Statement. JAMA. 2021;under review.

2. Skrivankova VW, Richmond RC, Woolf BAR, Davies NM, Swanson SA, VanderWeele TJ, et al. Strengthening the Reporting of Observational Studies in Epidemiology using Mendelian Randomisation (STROBE-MR): Explanation and Elaboration. BMJ. 2021;375:n2233.
